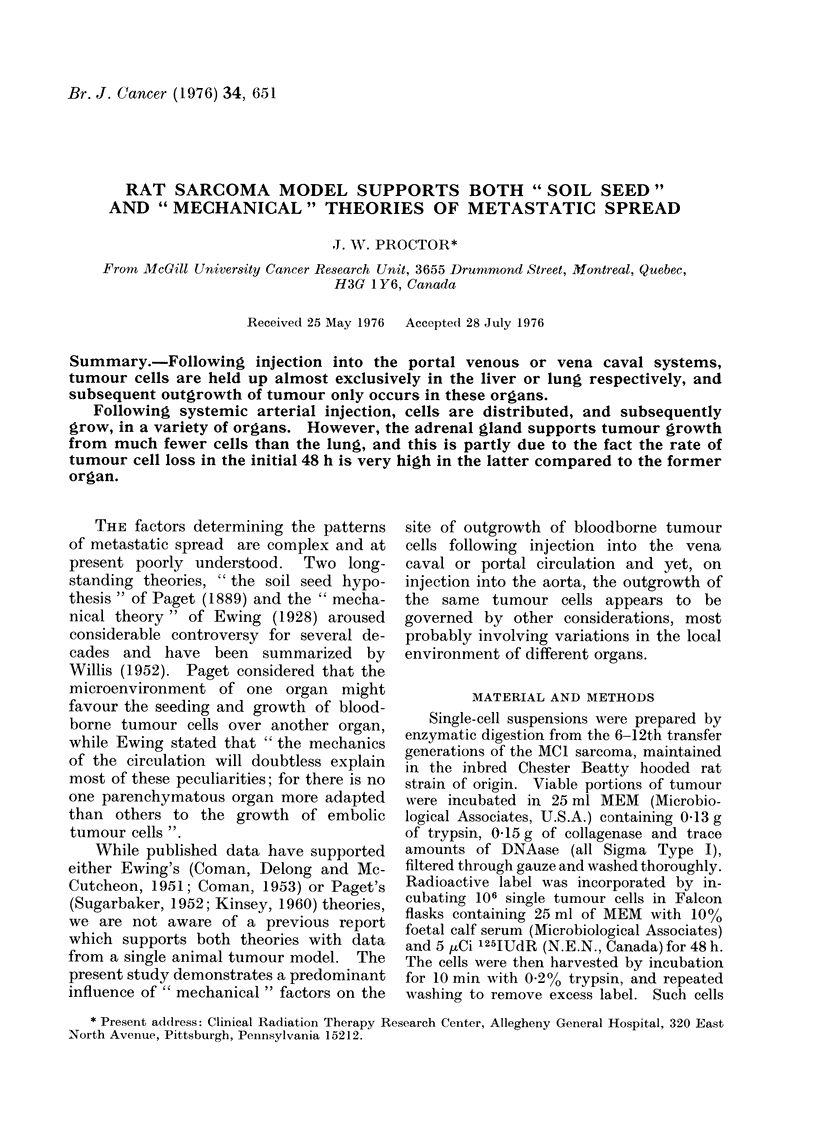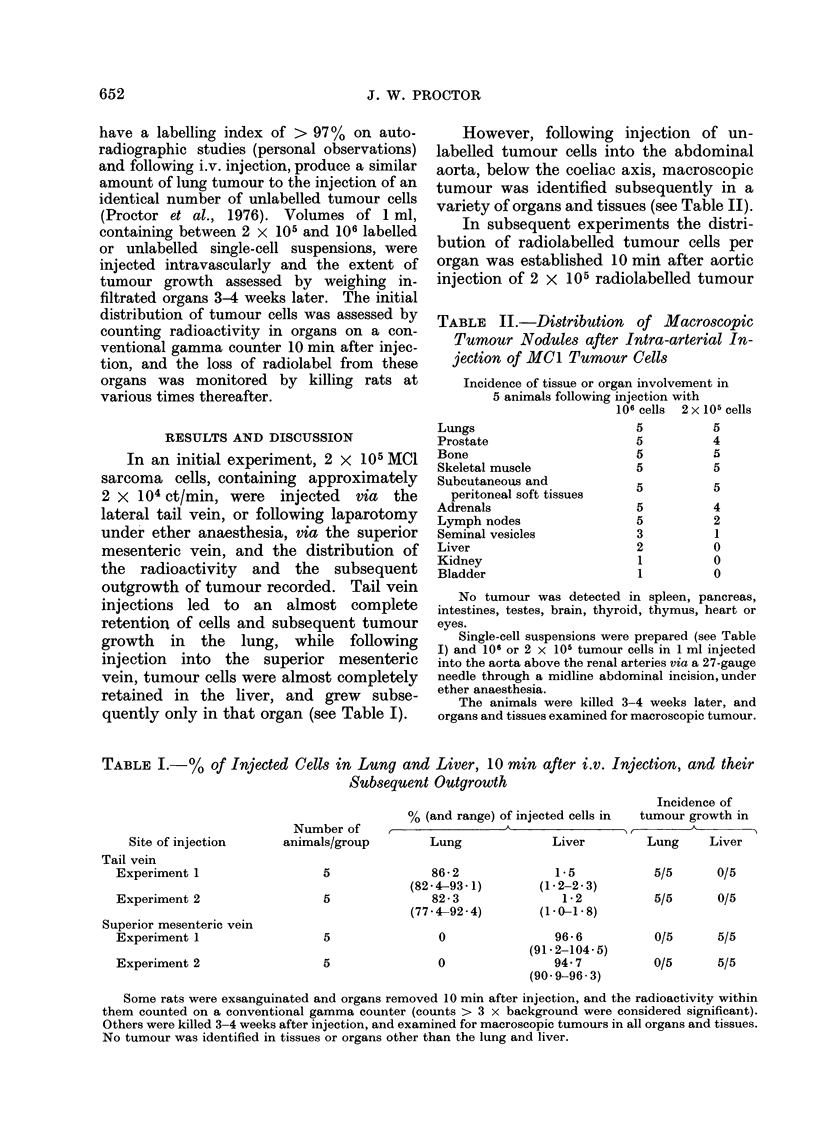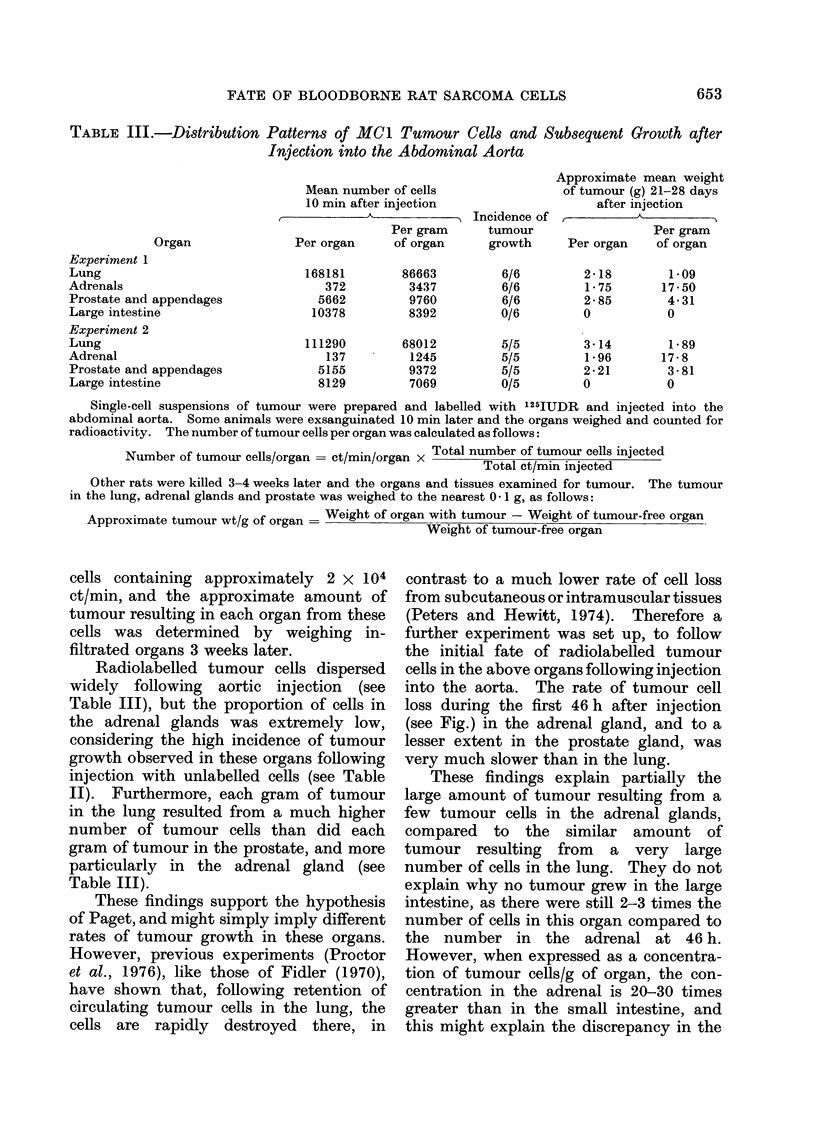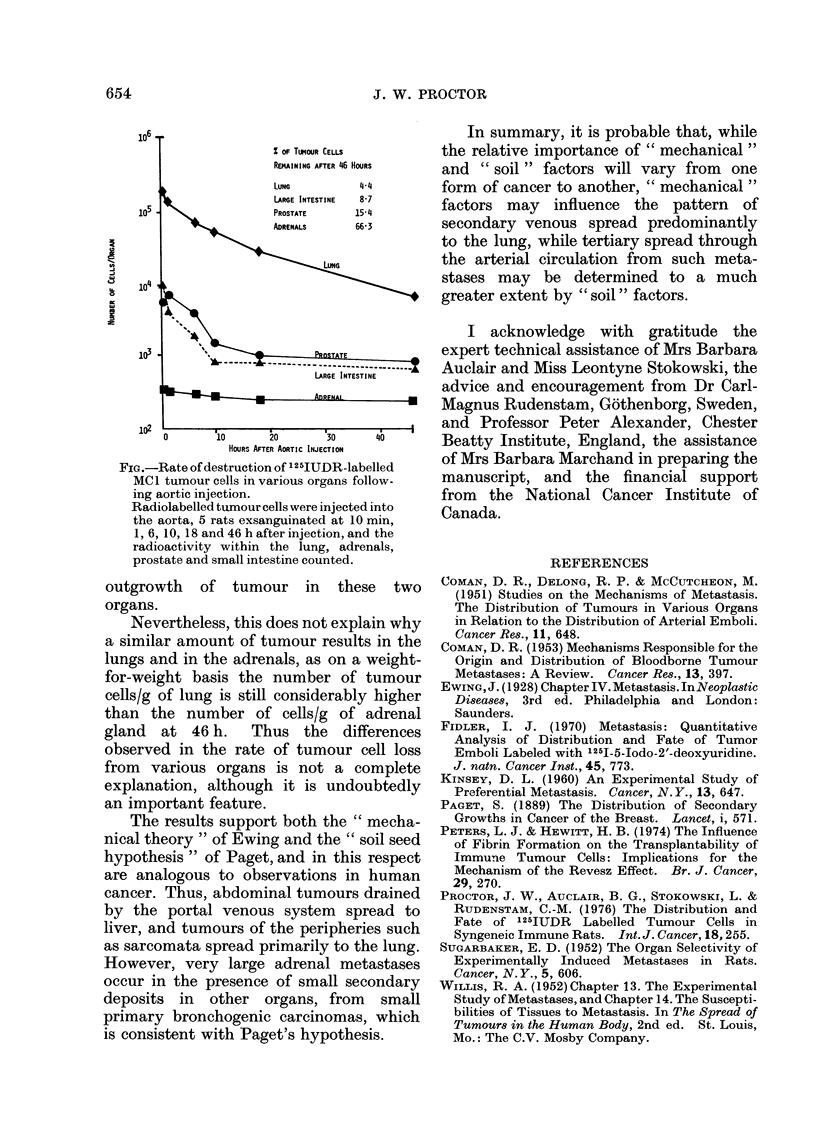# Rat sarcoma model supports both "soil seed" and "mechanical" theories of metastatic spread.

**DOI:** 10.1038/bjc.1976.227

**Published:** 1976-12

**Authors:** J. W. Proctor

## Abstract

Following injection into the portal venous or vena caval systems, tumour cells are held up almost exclusively in the liver or lung respectively, and subsequent outgrowth of tumour only occurs in these organs. Following systemic arterial injection, cells are distributed, and subsequently grow, in a variety of organs. However, the adrenal gland supports tumour growth from much fewer cells than the lung, and this is partly due to the fact the rate of tumour cell loss in the initial 48 h is very high in the latter compared to the former organ.


*Br. J. Cancer* (1976) **34**, 651

### RAT SARCOMA MODEL SUPPORTS BOTH "SOIL SEED" AND "MECHANICAL" THEORIES OF METASTATIC SPREAD

J. W. PROCTOR*

*From McGill University Cancer Research Unit, 3655 Drummond Street, Montreal, Quebec, H3G 1Y6, Canada*

Received 25 May 1976 Accepted 28 July 1976

**Summary.—Following injection into the portal venous or vena caval systems, tumour cells are held up almost exclusively in the liver or lung respectively, and subsequent outgrowth of tumour only occurs in these organs.**

**Following systemic arterial injection, cells are distributed, and subsequently grow, in a variety of organs. However, the adrenal gland supports tumour growth from much fewer cells than the lung, and this is partly due to the fact the rate of tumour cell loss in the initial 48 h is very high in the latter compared to the former organ.**

THE factors determining the patterns of metastatic spread are complex and at present poorly understood. Two long-standing theories, "the soil seed hypothesis" of Paget (1889) and the "mechanical theory" of Ewing (1928) aroused considerable controversy for several decades and have been summarized by Willis (1952). Paget considered that the microenvironment of one organ might favour the seeding and growth of bloodborne tumour cells over another organ, while Ewing stated that "the mechanics of the circulation will doubtless explain most of these peculiarities; for there is no one parenchymatous organ more adapted than others to the growth of embolic tumour cells".

While published data have supported either Ewing's (Coman, Delong and McCutcheon, 1951; Coman, 1953) or Paget's (Sugarbaker, 1952; Kinsey, 1960) theories, we are not aware of a previous report which supports both theories with data from a single animal tumour model. The present study demonstrates a predominant influence of "mechanical" factors on the site of outgrowth of bloodborne tumour cells following injection into the vena caval or portal circulation and yet, on injection into the aorta, the outgrowth of the same tumour cells appears to be governed by other considerations, most probably involving variations in the local environment of different organs.

### MATERIAL AND METHODS

Single-cell suspensions were prepared by enzymatic digestion from the 6–12th transfer generations of the MC1 sarcoma, maintained in the inbred Chester Beatty hooded rat strain of origin. Viable portions of tumour were incubated in 25 ml MEM (Microbiological Associates, U.S.A.) containing 0·13 g of trypsin, 0·15 g of collagenase and trace amounts of DNAase (all Sigma Type I), filtered through gauze and washed thoroughly. Radioactive label was incorporated by incubating $10^6$ single tumour cells in Falcon flasks containing 25 ml of MEM with 10% foetal calf serum (Microbiological Associates) and 5 $\mu$Ci $^{125}$IUdR (N.E.N., Canada) for 48 h. The cells were then harvested by incubation for 10 min with 0·2% trypsin, and repeated washing to remove excess label. Such cells

* Present address: Clinical Radiation Therapy Research Center, Allegheny General Hospital, 320 East North Avenue, Pittsburgh, Pennsylvania 15212.

have a labelling index of > 97% on autoradiographic studies (personal observations) and following i.v. injection, produce a similar amount of lung tumour to the injection of an identical number of unlabelled tumour cells (Proctor *et al.*, 1976). Volumes of 1 ml, containing between $2 \times 10^5$ and $10^6$ labelled or unlabelled single-cell suspensions, were injected intravascularly and the extent of tumour growth assessed by weighing infiltrated organs 3–4 weeks later. The initial distribution of tumour cells was assessed by counting radioactivity in organs on a conventional gamma counter 10 min after injection, and the loss of radiolabel from these organs was monitored by killing rats at various times thereafter.

### RESULTS AND DISCUSSION

In an initial experiment, $2 \times 10^5$ MCl sarcoma cells, containing approximately $2 \times 10^4$ ct/min, were injected *via* the lateral tail vein, or following laparotomy under ether anaesthesia, *via* the superior mesenteric vein, and the distribution of the radioactivity and the subsequent outgrowth of tumour recorded. Tail vein injections led to an almost complete retention of cells and subsequent tumour growth in the lung, while following injection into the superior mesenteric vein, tumour cells were almost completely retained in the liver, and grew subsequently only in that organ (see Table I).

However, following injection of unlabelled tumour cells into the abdominal aorta, below the coeliac axis, macroscopic tumour was identified subsequently in a variety of organs and tissues (see Table II).

In subsequent experiments the distribution of radiolabelled tumour cells per organ was established 10 min after aortic injection of $2 \times 10^5$ radiolabelled tumour

TABLE II.—*Distribution of Macroscopic Tumour Nodules after Intra-arterial Injection of MC*1 *Tumour Cells*

| | Incidence of tissue or organ involvement in 5 animals following injection with | |
|---|---|---|
| | $10^6$ cells | $2 \times 10^5$ cells |
| Lungs | 5 | 5 |
| Prostate | 5 | 4 |
| Bone | 5 | 5 |
| Skeletal muscle | 5 | 5 |
| Subcutaneous and peritoneal soft tissues | 5 | 5 |
| Adrenals | 5 | 4 |
| Lymph nodes | 5 | 2 |
| Seminal vesicles | 3 | 1 |
| Liver | 2 | 0 |
| Kidney | 1 | 0 |
| Bladder | 1 | 0 |

No tumour was detected in spleen, pancreas, intestines, testes, brain, thyroid, thymus, heart or eyes.

Single-cell suspensions were prepared (see Table I) and $10^6$ or $2 \times 10^5$ tumour cells in 1 ml injected into the aorta above the renal arteries *via* a 27-gauge needle through a midline abdominal incision, under ether anaesthesia.

The animals were killed 3–4 weeks later, and organs and tissues examined for macroscopic tumour.

TABLE I.—% *of Injected Cells in Lung and Liver,* 10 *min after i.v. Injection, and their Subsequent Outgrowth*

| Site of injection | Number of animals/group | % (and range) of injected cells in: Lung | Liver | Incidence of tumour growth in: Lung | Liver |
|---|---|---|---|---|---|
| Tail vein | | | | | |
| Experiment 1 | 5 | 86·2 (82·4–93·1) | 1·5 (1·2–2·3) | 5/5 | 0/5 |
| Experiment 2 | 5 | 82·3 (77·4–92·4) | 1·2 (1·0–1·8) | 5/5 | 0/5 |
| Superior mesenteric vein | | | | | |
| Experiment 1 | 5 | 0 | 96·6 (91·2–104·5) | 0/5 | 5/5 |
| Experiment 2 | 5 | 0 | 94·7 (90·9–96·3) | 0/5 | 5/5 |

Some rats were exsanguinated and organs removed 10 min after injection, and the radioactivity within them counted on a conventional gamma counter (counts > 3 × background were considered significant). Others were killed 3–4 weeks after injection, and examined for macroscopic tumours in all organs and tissues. No tumour was identified in tissues or organs other than the lung and liver.

TABLE III.—*Distribution Patterns of MC1 Tumour Cells and Subsequent Growth after Injection into the Abdominal Aorta*

| Organ | Mean number of cells 10 min after injection: Per organ | Mean number of cells 10 min after injection: Per gram of organ | Incidence of tumour growth | Approximate mean weight of tumour (g) 21–28 days after injection: Per organ | Approximate mean weight of tumour (g) 21–28 days after injection: Per gram of organ |
|---|---|---|---|---|---|
| *Experiment* 1 | | | | | |
| Lung | 168181 | 86663 | 6/6 | 2·18 | 1·09 |
| Adrenals | 372 | 3437 | 6/6 | 1·75 | 17·50 |
| Prostate and appendages | 5662 | 9760 | 6/6 | 2·85 | 4·31 |
| Large intestine | 10378 | 8392 | 0/6 | 0 | 0 |
| *Experiment* 2 | | | | | |
| Lung | 111290 | 68012 | 5/5 | 3·14 | 1·89 |
| Adrenal | 137 | 1245 | 5/5 | 1·96 | 17·8 |
| Prostate and appendages | 5155 | 9372 | 5/5 | 2·21 | 3·81 |
| Large intestine | 8129 | 7069 | 0/5 | 0 | 0 |

Single-cell suspensions of tumour were prepared and labelled with $^{125}$IUDR and injected into the abdominal aorta. Some animals were exsanguinated 10 min later and the organs weighed and counted for radioactivity. The number of tumour cells per organ was calculated as follows:

$$\text{Number of tumour cells/organ} = \text{ct/min/organ} \times \frac{\text{Total number of tumour cells injected}}{\text{Total ct/min injected}}$$

Other rats were killed 3–4 weeks later and the organs and tissues examined for tumour. The tumour in the lung, adrenal glands and prostate was weighed to the nearest 0·1 g, as follows:

$$\text{Approximate tumour wt/g of organ} = \frac{\text{Weight of organ with tumour} - \text{Weight of tumour-free organ}}{\text{Weight of tumour-free organ}}$$

cells containing approximately $2 \times 10^4$ ct/min, and the approximate amount of tumour resulting in each organ from these cells was determined by weighing infiltrated organs 3 weeks later.

Radiolabelled tumour cells dispersed widely following aortic injection (see Table III), but the proportion of cells in the adrenal glands was extremely low, considering the high incidence of tumour growth observed in these organs following injection with unlabelled cells (see Table II). Furthermore, each gram of tumour in the lung resulted from a much higher number of tumour cells than did each gram of tumour in the prostate, and more particularly in the adrenal gland (see Table III).

These findings support the hypothesis of Paget, and might simply imply different rates of tumour growth in these organs. However, previous experiments (Proctor *et al.*, 1976), like those of Fidler (1970), have shown that, following retention of circulating tumour cells in the lung, the cells are rapidly destroyed there, in contrast to a much lower rate of cell loss from subcutaneous or intramuscular tissues (Peters and Hewitt, 1974). Therefore a further experiment was set up, to follow the initial fate of radiolabelled tumour cells in the above organs following injection into the aorta. The rate of tumour cell loss during the first 46 h after injection (see Fig.) in the adrenal gland, and to a lesser extent in the prostate gland, was very much slower than in the lung.

These findings explain partially the large amount of tumour resulting from a few tumour cells in the adrenal glands, compared to the similar amount of tumour resulting from a very large number of cells in the lung. They do not explain why no tumour grew in the large intestine, as there were still 2–3 times the number of cells in this organ compared to the number in the adrenal at 46 h. However, when expressed as a concentration of tumour cells/g of organ, the concentration in the adrenal is 20–30 times greater than in the small intestine, and this might explain the discrepancy in the

FIG.—Rate of destruction of $^{125}$IUDR-labelled MC1 tumour cells in various organs following aortic injection.
Radiolabelled tumour cells were injected into the aorta, 5 rats exsanguinated at 10 min, 1, 6, 10, 18 and 46 h after injection, and the radioactivity within the lung, adrenals, prostate and small intestine counted.

outgrowth of tumour in these two organs.

Nevertheless, this does not explain why a similar amount of tumour results in the lungs and in the adrenals, as on a weight-for-weight basis the number of tumour cells/g of lung is still considerably higher than the number of cells/g of adrenal gland at 46 h. Thus the differences observed in the rate of tumour cell loss from various organs is not a complete explanation, although it is undoubtedly an important feature.

The results support both the " mechanical theory " of Ewing and the " soil seed hypothesis " of Paget, and in this respect are analogous to observations in human cancer. Thus, abdominal tumours drained by the portal venous system spread to liver, and tumours of the peripheries such as sarcomata spread primarily to the lung. However, very large adrenal metastases occur in the presence of small secondary deposits in other organs, from small primary bronchogenic carcinomas, which is consistent with Paget's hypothesis.

In summary, it is probable that, while the relative importance of " mechanical " and " soil " factors will vary from one form of cancer to another, " mechanical " factors may influence the pattern of secondary venous spread predominantly to the lung, while tertiary spread through the arterial circulation from such metastases may be determined to a much greater extent by " soil " factors.

I acknowledge with gratitude the expert technical assistance of Mrs Barbara Auclair and Miss Leontyne Stokowski, the advice and encouragement from Dr Carl-Magnus Rudenstam, Göthenborg, Sweden, and Professor Peter Alexander, Chester Beatty Institute, England, the assistance of Mrs Barbara Marchand in preparing the manuscript, and the financial support from the National Cancer Institute of Canada.

### REFERENCES

COMAN, D. R., DELONG, R. P. & MCCUTCHEON, M. (1951) Studies on the Mechanisms of Metastasis. The Distribution of Tumours in Various Organs in Relation to the Distribution of Arterial Emboli. *Cancer Res.*, **11**, 648.

COMAN, D. R. (1953) Mechanisms Responsible for the Origin and Distribution of Bloodborne Tumour Metastases: A Review. *Cancer Res.*, **13**, 397.

EWING, J. (1928) Chapter IV. Metastasis. In *Neoplastic Diseases*, 3rd ed. Philadelphia and London: Saunders.

FIDLER, I. J. (1970) Metastasis: Quantitative Analysis of Distribution and Fate of Tumor Emboli Labeled with $^{125}$I-5-Iodo-2′-deoxyuridine. *J. natn. Cancer Inst.*, **45**, 773.

KINSEY, D. L. (1960) An Experimental Study of Preferential Metastasis. *Cancer, N.Y.*, **13**, 647.

PAGET, S. (1889) The Distribution of Secondary Growths in Cancer of the Breast. *Lancet*, i, 571.

PETERS, L. J. & HEWITT, H. B. (1974) The Influence of Fibrin Formation on the Transplantability of Immune Tumour Cells: Implications for the Mechanism of the Revesz Effect. *Br. J. Cancer*, **29**, 270.

PROCTOR, J. W., AUCLAIR, B. G., STOKOWSKI, L. & RUDENSTAM, C.-M. (1976) The Distribution and Fate of $^{125}$IUDR Labelled Tumour Cells in Syngeneic Immune Rats. *Int. J. Cancer*, **18**, 255.

SUGARBAKER, E. D. (1952) The Organ Selectivity of Experimentally Induced Metastases in Rats. *Cancer, N.Y.*, **5**, 606.

WILLIS, R. A. (1952) Chapter 13. The Experimental Study of Metastases, and Chapter 14. The Susceptibilities of Tissues to Metastasis. In *The Spread of Tumours in the Human Body*, 2nd ed. St. Louis, Mo.: The C.V. Mosby Company.